# A gene therapy induced emphysema model and the protective role of stem cells

**DOI:** 10.1186/s13000-014-0195-7

**Published:** 2014-11-14

**Authors:** Paul Zarogoulidis, Wolfgang Hohenforst-Schmidt, Haidong Huang, Despoina Sahpatzidou, Lutz Freitag, Leonidas Sakkas, Aggeliki Rapti, Ioannis Kioumis, Georgia Pitsiou, Kokkona Kouzi-Koliakos, Anna Papamichail, Antonis Papaiwannou, Theodora Tsiouda, Kosmas Tsakiridis, Konstantinos Porpodis, Sofia Lampaki, John Organtzis, Andreas Gschwendtner, Konstantinos Zarogoulidis

**Affiliations:** Pulmonary Department, “G. Papanikolaou” General Hospital, Aristotle University of Thessaloniki, Thessaloniki, Greece; II Medical Department, “Coburg” Regional Department, University of Wuerzburg, Coburg, Germany; Department of Respiratory Diseases, Changhai Hospital/First Affiliated Hospital of the Second Military Medical University, Shanghai, China; Experimental Animal Laboratory, “Theiageneio” Anticancer Hospital, Thessaloniki, Greece; Department of Interventional Pneumology, Ruhrlandklinik, West German Lung Center, University Hospital, University Duisburg-Essen, Essen, Germany; Pathology Department, “G. Papanikolaou” General Hospital, Thessaloniki, Greece; Pulmonary Department, “Sotiria” Hospital of Chest Diseases, Athens, Greece; Department of Histology Embryology, School of Medicine, Aristotle University of Thessaloniki, Thessaloniki, Greece; Internal Medicine Department, “Thegenio” Anticancer Hospital, Thessaloniki, Greece; Cardiothoracic Surgery Department, Saint “Luke” Private Hospital, Thessaloniki, Panorama Greece; Pathology Department, Hospital of Amberg, Amberg, Germany

**Keywords:** Emphysema, Stem cells, Gene therapy, DDMC

## Abstract

**Background:**

Chronic obstructive pulmonary disease presents with two different phenotypes: chronic bronchitis and emphysema with parenchymal destruction. Decreased expression of vascular endothelial growth factor and increased endothelial cell apoptosis are considered major factors for emphysema. Stem cells have the ability of vascular regeneration and function as a repair mechanism for the damaged endothelial cells. Currently, minimally invasive interventional procedures such as placement of valves, bio-foam or coils are performed in order to improve the disturbed mechanical function in emphysema patients. However, these procedures cannot restore functional lung tissue. Additionally stem cell instillation into the parenchyma has been used in clinical studies aiming to improve overall respiratory function and quality of life.

**Methods:**

In our current experiment we induced emphysema with a DDMC non-viral vector in BALBC mice and simultaneously instilled stem cells testing the hyposthesis that they might have a protective role against the development of emphysema. The mice were divided into four groups: a) control, b) 50.000 cells, c) 75.000 and d) 100.000 cells.

**Results:**

Lung pathological findings revealed that all treatment groups had less damage compared to the control group. Additionally, we observed that emphysema lesions were less around vessels in an area of 10 μm.

**Conclusions:**

Our findings indicate that stem cell instillation can have a regenerative role if applied upon a tissue scaffold with vessel around.

**Virtual Slides:**

The virtual slide(s) for this article can be found here: http://www.diagnosticpathology.diagnomx.eu/vs/13000_2014_195

## Background

Chronic obstructive pulmonary disease (COPD) is characterized by chronic inflammation and progressive destruction of lung parenchyma. Currently there are no interventions able to reverse or even stop disease progression. Transplantation is the only option to regain functional tissue. Full medical treatment and smoking cessation are able to slow the progression of emphysema, improve quality of life and prolong survival [1]. Surgical lung volume reduction reduces hyperinflation and improves overall functional status although lung tissue is sacrificed. This approach is considered feasible for patients with predominantly upper lobe emphysema. In order to avoid surgery, minimally invasive interventional bronchoscopy techniques have been developed. The application of endobronchial valves, coils and bio-foam have been recently added as arrows in the quiver for emphysema treatment [2-4]. Despite all pharmaceutical or interventional treatment modalities the loss of lung tissue and remodeling continues [5,6]. Chronic obstructive pulmonary disease involves airways of all generations, the alveoli (<2 mm) and the tissue beyond. The major dysfunction observed clinically is the change of airflow parameters due to the loss of elastic lung recoil. Furthermore; endothelial dysfunction and vascular remodeling are initiated by vascular endothelial growth factor (VEGF)-mediated apoptosis [7]. Inflammatory response is also initiated by exposure to environmental factors such as; air pollution and cigarette smoking. Key factors in the destructive cascades are proteolytic substances and oxidant stress [8,9]. The irreversible process is further accelerated by frequent exacerbations. Despite the removal of the trigger factor, the inflammatory cells (neutrophils, macrophages and lymphocytes) still remain in large numbers within the respiratory airways and stimulate inflammation, remodeling and destruction, due to nature of the continuous exposure [10,11]. The airway epithelium has slow proliferative and renewal capability, unless injured [12]. While differentiated cells cannot compensate for the loss of complex lung tissue destroyed by the above outline processes, stem cells may have these capabilities. Stem cells have the ability of self-renewal and through trophic units they are mobilized and attracted to the site of damage [13]. Stem cells can be classified according to their potency to differentiate, in: *unipotent* stem cells, able to produce only one cell type, *multipotent* cells able to form all cells of one particular lineage, *pluripotent* stem cells capable of differentiating into any of the embryonic germ layers [14]. Finally, there are *totipotent* cells that can give rise to an entire organism. This property is retained in mammals by the zygote and up to at least 4-cell stage embryo [15-17].

Stem cells are also classified in *embryonic* stem cells and *adult* (ASCs) or non embryonic stem cells. The pluripotent embryonic stem cells derived from the inner cell mass of the blastocyst have the unique capacity to generate any cell of the body. Additionally they can stay as undifferentiated cell type for prolonged period of time in culture [16,18,19]. Adult stem cells (ACSs) have self-renewal capability, they are multipotent cells obtained from adult tissues.

Because of their unique characteristics, self-renewing and multilineage differentiation, stem cells and especially mesemchymal stem cells MSCs, are promising candidates for potential therapeutic uses in regenerative medicine, cell-based therapy, dentistry and tissue engineering [20,21]. Stem cells plasticity and regeneration ability has rend them a great promise for the treatment of a variety of diseases such as cardiovascular diseases, heart failure, diabetes, liver diseases, stroke, Parkinson’s and Huntinghton disease and cirrhosis [20,22,23]. Moreover; they poses the ability to modulate immune system responses by modulating the immunosuppressive effect of B and T lymphocytes and therefore they have been used as a possible treatment for autoimmune diseases, systemic sclerosis rheumatoid arthritis, systemic lupus erythematosus, and autoimmune encephalomyelitis [24-26]. Currently, many research labs conduct animal and human studies investigating the protective effect of stem cells in the lung parenchyma [27-34]. We had recently developed an animal model of induced lung emphysema by a non-viral vector DDMC [35]. In the present study we investigated whether adult stem cells administered by a sprayer probe after emphysema induction might have a protective or even therapeutic effect.

## Methods

### Aerosol administration

#### Non-viral vector

The non-viral vector was purchased from Ryujyu science corporation, Seto-City, Japan by PZ and WHS under the contract EG179806600JP. The non-viral vector has the following characteristics; fast and easy procedure, stable for autoclaving sterilization at 121°C for 15 minutes, broad peak performance, applicable in high-throughput-screening (HTS), no serum inhibition, broad cell line range, best results with siRNA applications, excellent reproducibility, low toxicity in comparison with DEAE-dextran, high efficiency by use of low DNA amounts, a high DNase protection facility by DNase degradation. Encouraged by our previous results [35] we chose this vector for this stem cell study.

#### Animals

Eighty BALBC mice age 7–8 weeks old, purchased from the “Theageneio” Anticancer Hospital. The Institute has the following authorization for production and experimentation of mice EL 25 BIO 011 and EL 25 BIO 013. The protocol was authorized by the Aristotle University of Thessaloniki. The mice included were isolated (1 per cage) in a temperature-controlled room on 12-hour light–dark cycle and were allowed free access to food and water. The 80 mice were divided in four groups receiving different amounts of stem cells: a) control group (only non viral vector administration), b) 50.000 stem cells plus non-viral vector, c) 75.000 stem cells plus non-viral vector, d) 100.000 stem cells plus non-viral vector.

#### Protocol

**Figure 1:**
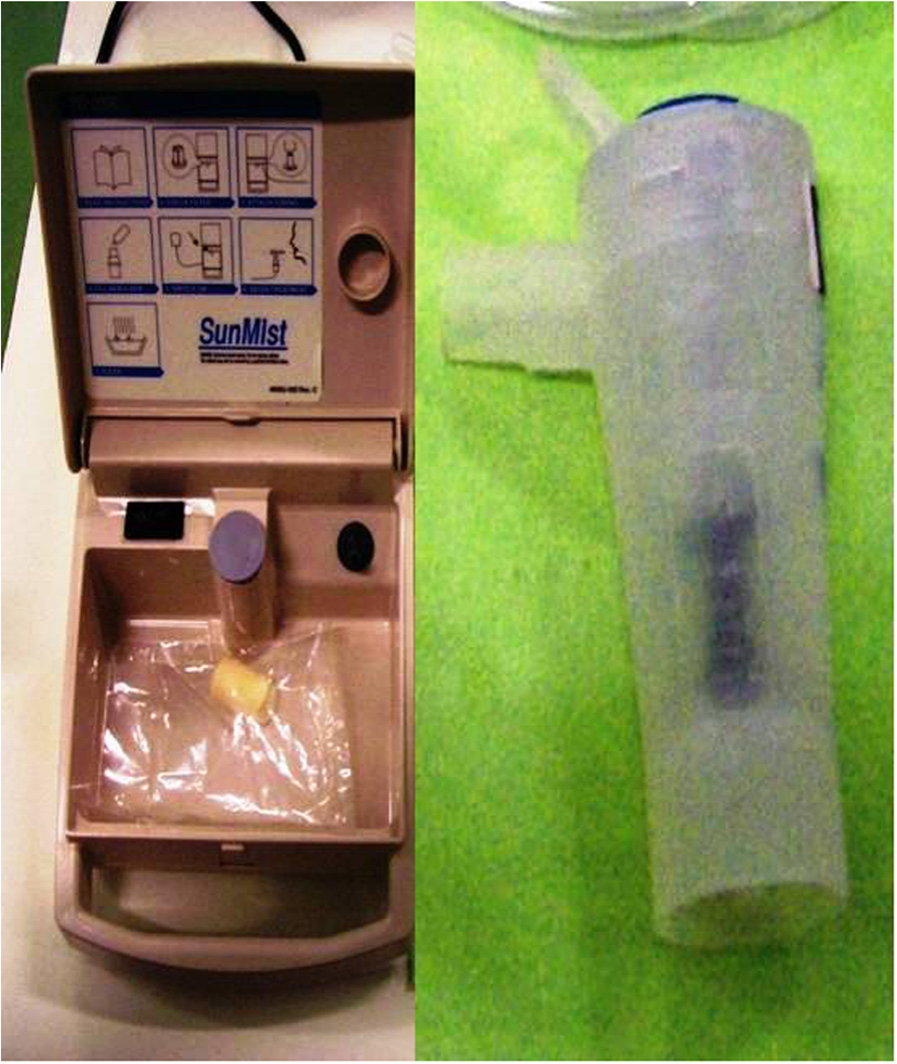
**Jet-Nebulizer sunmist with a residual cup of maximum load 10 ml.**

**Figure 2:**
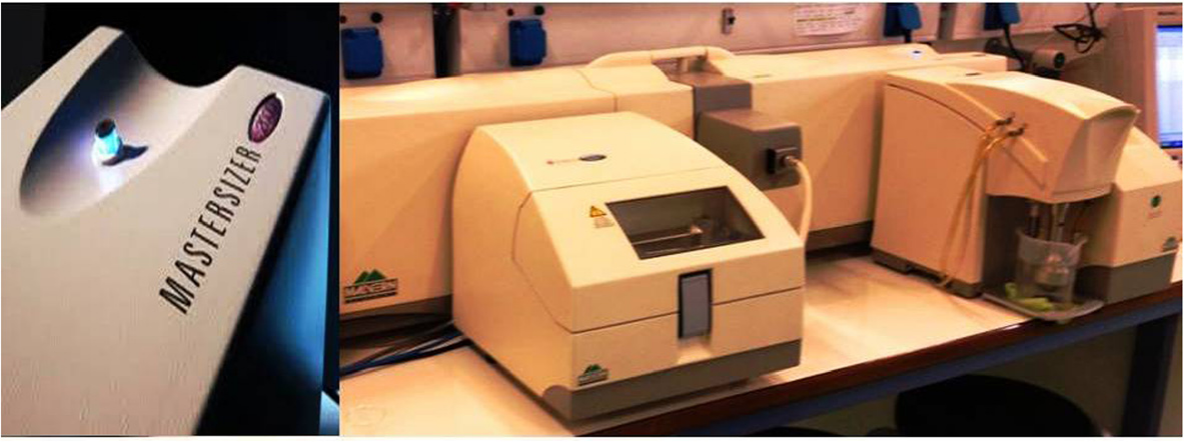
**Equipment Mastersizer 2000 for aerosol droplet measurement.**

The vector was delivered in 1 ml and was diluted with 10 ml of 5% glucose solution (11 ml solution in total), as previously published [35]. The mixture was vortexed gently in order to homogenize. A Sunmist® compressor nebulizer (6 liters/minute and 35 psi) was used to aerosolize 1 ml/11 ml in 5 minutes for each mouse (Figure 1). Each subject received 1 ml prior to stem cell instillation. The size distribution of the solution droplets was determined using a Malvern Mastersizer 2000 laser scattering apparatus (Malvern, Worcestershire, UK) equipped with a Scirocco module (Malvern, Worcestershire, UK) (Figure 2). The latter was modified as for the dispersion to be sprayed directly into the sample chamber, normal to the laser beam. A refractive index of 1.33 was used for the dispersed phase. A minimum of three repetitions were held for each treatment. The mass median aerodynamic diameter was 3.9 μm. It has to be noted that the dilution directions were followed as indicated by the company Ryujyu Science, however; a different nebulization system was used. We administered nebulisation of 4–10 ml 5% glucose solution with an ultrasound device (2 MHz) over a period of 15–30 min. The aerosol was delivered to the lungs of the mice in a nose only sealed plastic cage. Details can been found in our previous publication [35].

#### Stem cells

**Figure 3:**
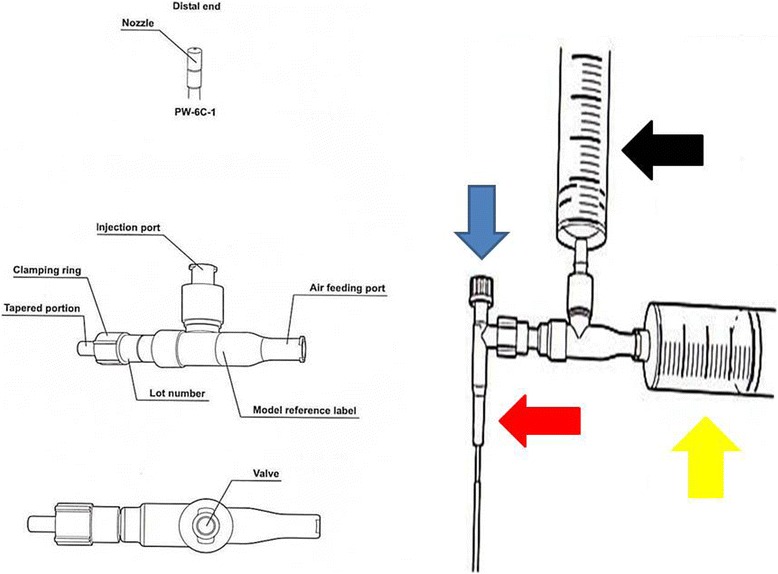
**Equipment for stem cell instillation (Olympus stylet probe).** Black arrow represents the syringe with the stem cells, yellow arrow represents the syringe working as the air inflator, red arrow represents the probe that enters the mouse airway and the blue arrow represents the metallic stylet within the probe which assists in the efficient deposition of the probe within the trachea of the mice.

Human Umbilical Cord Mesenchymal Cells (HUCMCs) isolated from Wharton’s Jelly was provided by Biohellenika Biotechnology Company. HUCMCs were plated in 6-well plates (BD Falcon) and cultured in Dulbecco’s modified Eagle medium supplemented with 10% fetal bovine serum, penicillin (100 IU/ml) and streptomycin (100 μg/ml). HUCMCs were grown to 60% confluency and then, transfected with 5 μg of DNA mixture encoding SB100x transponase and pT2-Venus-neo transposon expression plasmids (1:9 ratio). Transfection was performed using Xfect™ reagent (Clontech), according to the manufacturer’s instructions. HUCMCs stably expressing both the fluorescent Venus and antibiotic-resistance neo genes were selected using 0.5 mg/ml G418. The stem cells were administered with an Olympus® spray catheter (with Stylet) PW-6C-1 (working channel 1050 mm and minimum channel size 2.mm). Two syringes were used; a) insulin (100unit loading) and b) 10 ml loading (Figure 3). For this protocol we used three groups; a) 50.000 cells, b) 75.000 cells and c) 100.000 cells.

### Pathology and imaging technique

Sections of 4 microns of lung tissue samples were cut using freezing microtome (Leica CM1900). A polarized light microscope (Zeiss) was used to examine unstained lung tissue sections on glass slides.

Moreover; the lung tissues were placed in labeled plasic cassettes and after overnight 18 hour schedule using an automatic tissue processor (Thermo Scientific Wxcelsior, ES Histokinette) sections of 4–5 microns were cut from paraffin blocks using a microtome (MICROM, HM340E). All the sections were stained with the heamatoxylin and eosin method.

## Results and discussion

**Figure 4:**
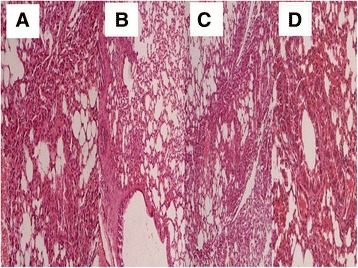
**Pathology specimen after stem cell administration.** 7 days after the first administration; **A)** control group, **B)** 50.000 stem cell group, **C)** 75.000 stem cell group and **D)** 100.000 stem cell group.

**Figure 5:**
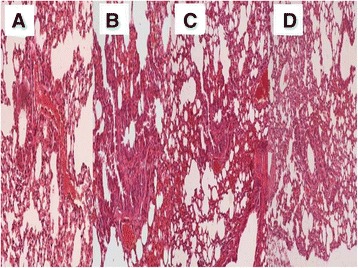
**Pathology specimen after stem cell administration.** 14 days after the first administration; **A)** control group, **B)** 50.000 stem cell group, **C)** 75.000 stem cell group and **D)** 100.000 stem cell group.

**Figure 6:**
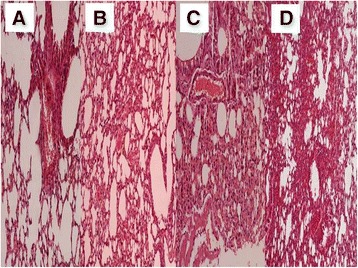
**Pathology specimen after stem cell administration.** 21 days after the first administration; **A)** control group, **B)** 50.000 stem cell group, **C)** 75.000 stem cell group and **D)** 100.000 stem cell group.

**Figure 7:**
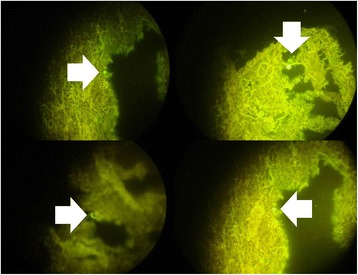
**White arrows indicate stem cells within alveoli.**

In order to properly evaluate the effect of the non-viral vector to the lung parenchyma and the effect of the stem cells in different concentrations we sacrificed animals at the following time points. At the end of the first week of the experiment (Figure 4), second week (Figure 5) and third and final week (21 days after the first aerosol-stem cell application) (Figure 6). We also present evidence with Figure 7 where the stem cells are displayed with fluorescence in dark background, the stem cells are within the alveoli, providing clear evidence that they distributed efficiently with our methodology. Our pathological findings indicate that the non-viral vector induced emphysema as previously [35] and that the higher the concentration of the instilled stem cells >50.000 cell population the lower the emphysema lesions observed. The stem cells did not reconstruct in any case alveoli or other type of tissue within the lung parenchyma, however; it seems that they acted as an anti-inflammatory factor.

## Conclusions

Chronic obstructive pulmonary disease and emphysema are known to be associated with air pollution and smoking habit [36]. COPD and emphysema reduce the respiratory capability of the subject and induce lung injury which in several situations are life threatening [37]. Currently several drugs such as inhaled bronchodilators, inhaled corticosteroids, phosphodiesterase-4 inhibitors and mucolytics are used as symptomatic therapy and blockage of inflammation [38-40]. Other drugs such as macrolides have been also used for control of COPD exacerbations by exploiting their anti-inflammatory properties [41]. It has been observed that during COPD exacerbation the circulating endothelial stem cells are not decreased, however; their population is abnormally high, probably during an effort for tissue repair and inflammation control [42,43]. Increased levels of vascular endothelial growth factor (VEGF) have been associated with increased levels of circulating endothelial stem cells [43]. It has been previously observed that mesenchymal stem cells protect cigarette smoke lung injury, by up-regulating vascular endothelial growth factor receptor 2 and tumor growth factor β-1, and down-regulating inflammatory response, cell apoptosis and excessive protease expression [27]. In laboratory model experiments, mesenchymal stem cells have been co-cultured with scaffolds. Producing functional parts of the respiratory system, provided evidence that these cells might be used for tissue repair in the airways [44]. The same observation has been made for other diseases of the respiratory system such pulmonary fibrosis, cystic fibrosis and pulmonary hypertension [45]. In vivo imaging of these cells has been previously accomplished during airway regeneration in mice [30]. It has been also shown that bone marrow cells could repair cigarette induced emphysema [28,46]. The same observation was made for stem cells derived from the adipose tissue [47]. However; there is an ongoing investigation regarding another stem cell population such as the bronchioalveolar stem cells, and how this population affects the initiation of a respiratory disease and whether it can reverse it [48]. Other researches use basal stem cells and investigate their role in epithelial homeostasis and remodeling [49]. Activation of satellite cells in the intercostals muscles has been also proposed as an additional method of assisting the respiratory function of COPD patients [50]. Based on previously presented data, clinical studies have been started, evaluating the potential tissue repair ability of stem cells by administering them in patients with copd [28,29,31]. Early results indicate that the stem cell administration is safe and that the respiratory function can be improved. Currently this treatment lacks of pathological evidence of whether it can truly regenerate alveoli units, or whether the improvement of the respiratory function is due to the anti-inflammatory property. It has been previously proposed that mesenchymal stem cells have the ability to regenerate tissue [51]. Even if these cells had unlimited capabilities, the main problem of an in vivo tissue engineering effort is to make a cell population create new healthy and functioning alveoli as in our case, or repair damaged ones. There are several possible approaches. One could create a scaffold with an extracellular matrix guiding stem cells to create the desired structure or one could bioengineer stem cell with the genetic information to create alveoli. Possibly a disease by disease case should be investigated. In our experiment we provide a novel model of emphysema. We found no pathological evidence that under these conditions, stem cells would create new alveoli tissue or any other structure of the respiratory system. However; image analysis proved that the stem cells assisted in down-regulating the inflammation of the trigger factor (DDMC non-viral vector). Lipopolysaccharides have been also recently used to induce emphysema [52]. The anti-inflammatory effect has been previously observed in other studies [27]. Major limitations of our study were firstly the lack of inflammatory marker evaluation however; tissue damage is observed to be inversely associated with the administration of Human Umbilical Cord Mesenchymal Cells (HUCMCs) population. Secondly we did not evaluate the survival of the groups, however; our objective was to present solid data (pathological) whether stem cells regenerate any kind of tissue formation within the lung parenchyma when a lung injury trigger factor exists. Moreover; we did not evaluate the population of stem cells deposited per mm^2^ of alveoli. It was impossible to know exactly the population of the stem cells instilled in the lungs and hoe of them were deposited in the alveoli since the mice in several occasions exhaled the stem cell compound due to cough. Additionally, less damage to the alveoli was observed next to vessels (<10 μm) probably, because as previously observed these cells are attract by vascular endothelial growth factors [53].

In our current work we investigated the protective effect of adult Human Umbilical Cord Mesenchymal Cells (HUCMCs) in a gene therapy induced emphysema model. We observed that any dosage from ≥50.000cells has a protective effect. The observation that we find most interesting is that around the vessels of the lung parenchyma in an area of no more than 10 μm the emphysema lesions were significantly reduced. Our conclusion based on pathological findings is that vessels transport the regenerative cells and are diffused through the vascular wall to the regional tissue and that a kind of scaffold is necessary for the stem cells to regenerate lung parenchyma constructions.
